# Cultural Differences in Donation Decision-Making

**DOI:** 10.1371/journal.pone.0138219

**Published:** 2015-09-15

**Authors:** Yan Wang, Yi-Yuan Tang, Jinjun Wang

**Affiliations:** 1 Interdisciplinary Center for Social and Behavioral Studies, Dongbei University of Finance and Economics, Dalian, Liaoning, China; 2 Department of Psychological Sciences, Texas Tech University, Lubbock, Texas, United States of America; 3 School of Electrical Engineering, Dalian University of Technology, Dalian, Liaoning, China; The Lieber Institute for Brain Development, UNITED STATES

## Abstract

Decisions to help those in need are essential for human development and survival. Previous studies have demonstrated the “identified effect”, in which one identifiable individual typically invokes stronger feelings of compassion and receives greater aid than statistical victim. However, this preference might be influenced by cultural differences. In the current study, Chinese respondents’ ratings of distress and sympathy and their willingness to contribute are greater for a group of sick children than an individual. In the U.S., greater willingness to help and sympathy are elicited by an identified victim in comparison with an unidentified one. The different results may demonstrate the importance of cultural differences when trying to understand people’s prosocial behavior.

## Introduction

What motivates people to help others? Previous research has indicated that the presence of identifiable victims in helping behavior, induces immediate spontaneous emotional responses that usually increase willingness to make personal sacrifices to provide aid [1,2]. In a study tested the differences between identifiable and non-identifiable victims in the U.S., people were more inclined to help an identifiable victim than a statistical victim [3]. Studies conducted in Israel also showed that a single identified victim elicited stronger emotions (sympathy and distress) and higher willingness to contribute (WTC) than an unidentified single victim, and the effect of identification did not extend to a group of people [4,5]. However, whether the identified effect is truly universal is unknown.

Social psychological research on helping behaviors highlighted the role of emotions as motivators in helping decisions [6]. The affective reactions to the victims may be an important determinant of the choice to offer help, which might partially account for differences in contributions. Previous studies have found that participants who read about a single identified victim rated their emotions higher than participants who read about an unidentified victim [3,4,7]. It has been posited that the discrepancy in giving toward identifiable and unidentified victims might be mediated by affect [8]. A recent research has indicated that the processing of information concerning individuals and groups might be different, and an intense emotional reaction would be lead when the processing of information is related to a single identified victim, which has been suggested as an explanation to the identifiable victim effect [9].

A growing body of research has indicated that people’s cognition, emotion and motivation might be influenced by culture [10–13]. People in different cultures may differ in the way they perceive the world, their preferences, judgments and decisions [14,15]. Specifically, cultural values or norms may cause differences in willingness to help across different cultures. Although helping behaviors are common and valued in both Western and Eastern cultures, the motivation for such behaviors might be different [11,16,17]. Previous research indicated that Americans tend to frame the decision to help more as one's personal choice, whereas Eastern Indians are likely to frame it as a matter of moral responsibility [18]. Moreover, it has been suggested that Western individualism is based on conception of personal rights, for example, the practice of an altruistic act is subject to an individual’s right of choice; whereas Confucian ethics in Chinese culture is premised on the conceptions of personal duties and social goals rather than on personal rights [19]. Thus, we are able to infer that these fundamental cross-cultural differences are likely reflected in the helping behavior. It makes sense that providing identifying details might not increase contributions for Chinese participants, since the identified information might affect people’s personal liking, but it seems not to influence personal duties and social goals.

Research examining cultural differences posits that Easterners and Westerners have different cognitive and reasoning styles [14,15,20]. It was found that central tendencies in contextual reasoning were different between Westerners (typically Americans) and Easterners (typically Chinese) [21]. Chinese were found to prefer dialectical or compromise resolutions while Americans tended to use a linear and analytic reasoning. Evidence also showed that Westerners were more capable of ignoring contextual information, while Easterners were more capable of incorporating contextual information [22]. Research showed that the Americans fixated more on a salient target object while the Chinese made more saccades to the background when measuring the eye movements during scene perception, suggesting Americans and Chinese may have divergent perceptual strategies [23]. Since Chinese participants are more likely to focus on a broad perceptual and conceptual field rather than salient focal information, the identified information (mainly identified by picture) might be not as important as it is in western results.

In this study, we choose China and the U.S. as a representative to eastern and western society to test the cultural influences on donation decision-making. Based on previous research, we hypothesize that (1) the results of American participants may replicate the identifiable victim effect that an identifed vicitim typically ecilits higher contributions and greater emotions than an unidentified one, however, (2) the identifiable victim effect may not be replicated in China, since the perceptual strategies and the motivation in helping behaviors are diverse between the two countries, and (3) the Chinese may show greater emotional reactions and WTC to a group of victims than an individual due to the personal duties and social goals. The analysis mainly focus on two aspects of helping behaviors: the willingness to contribute and evoked emotions (sympathy and distress). Our study could provide the evidence of cultural differences for better understanding the prosocial behavior.

## Materials and Methods

Two hundred and eight Chinese undergraduates in China and 163 American college students in the U.S. volunteered to participate in this study. Participants in both groups were born and lived in their own countries. And the two groups have similar gender structure without statistical significance. Each participant provided written informed consent and received the same value of monetary compensation for their time and effort. The study was approved by the Institutional Review Board of Dalian University of Technology.

Participants were randomly assigned to one of four groups in a 2 (one vs. eight sick children) X 2 (identified vs. unidentified) experimental design as Kogut and Ritov did [4], and the sick child was [children were] always from the same cultural group as each participant. All participants read about a sick child [eight children] with leukemia whose life is [lives are] in danger. They were informed that an expensive medicine may save the life of the victim/s. In the identified condition, the ages, pictures and names of the victim/s were provided respectively in China and the U.S. The same eight children's pictures of each cultural group were used for identification of the group (using a group portrait), and the single victims (using the children from the group portrait) respectively in each cultural group. Each individual child was presented nearly an equal number of times in the identified single victim condition. After reading this description, Chinese participants read the following question in Chinese: “Imagine you had one hundred Yuan (about 15 $), how much (if any) of it would you donate to buy the medicine for the child [children] presented before?” Similarly, corresponding question conducted in the U.S. was in English: “Imagine you had one hundred dollars, how much (if any) of it would you donate to buy the medicine for the child [children] presented before?” Next, all participants were asked to rate their feelings of distress and sympathy both on a 7-point scale (ranging from “not at all” to “very much”) using two sentences as Kogut and Ritov did [4]. The sentence examining feelings of distress was: “After reading the child’s [children’s] story I felt worried, upset and sad.” The next sentence examining feelings of sympathy read: “I felt sympathy and compassion towards the sick child [children].”

## Results

Mean ratings of willingness to contribute in each condition were calculated respectively from the two cultural groups (reported in Table 1). Since no significant difference was found in WTC or emotional ratings for the eight different children in each cultural group (*F*(7,46) = 0.19, *p* = .99, *F*(7,32) = 0.58, *p* = .77 for Chinese and American WTC, respectively; *F*(7,46) = 0.89, *p* = .52, *F*(7,32) = 0.33, *p* = .94 for Chinese and American distress, respectively; *F*(7,46) = 0.30, *p* = .95, *F*(7,32) = 0.77, *p* = .62 for Chinese and American sympathy, respectively), we presented the average across all eight in the single identified victim condition in Chinese and American results respectively. As the WTC scores did not distribute normally (Normality test was performed on the WTC scores with one-sample Kolmogorov–Smirnov Test, p < .001), the WTC scores were log-transformed for the further analysis [7].

**Table 1:** Descriptive statistics of willingness to contribute (WTC) for both Chinese and American samples, computed separately for each experimental condition.

WTC	Single		Group of eight	
	Identified	Unidentified	Identified	Unidentified
Chinese (¥)				
N	54	49	51	54
Mean	27.41	30.00	58.24	49.63
SD	21.21	24.15	34.51	33.48
American ($)				
N	40	40	41	42
Mean	35.25	20.75	36.34	30.00
SD	27.83	20.56	27.91	21.86

### The WTC scores

**Fig 1:**
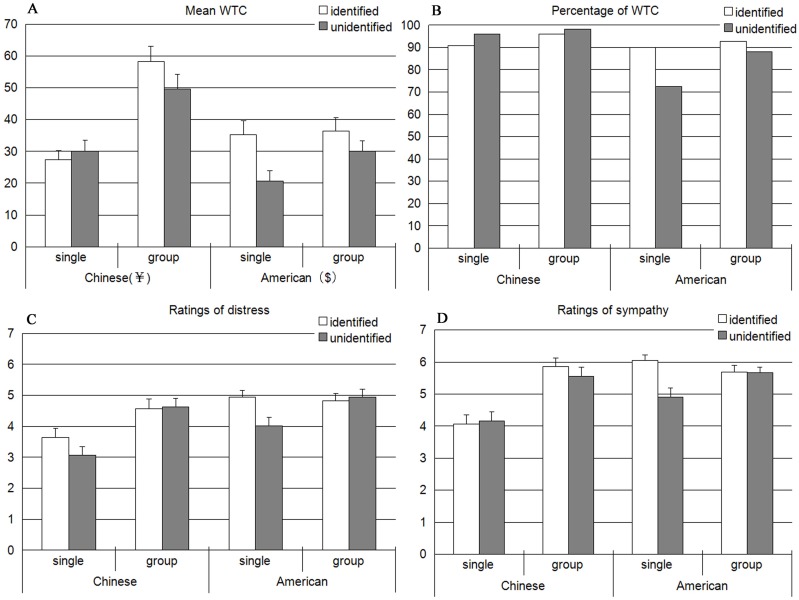
(A) Mean willingness to contribute, (B) Mean percentage of contributors, (C) Mean ratings of distress and (D) mean ratings of sympathy, for Chinese and American samples, as a function of singularity and identification of the victims. Error bars represent standard errors of the mean.

We analyzed the transformed WTC scores in the context of a 2 (sample: U.S. vs. China) X 2 (singularity: single vs. a group of eight victims) X 2 (identification: identified vs. unidentified) factorial design. Result showed a significant singularity effect, F(1,363) = 18.35, p < .001, and a marginally significant main effect of identification, F(1,363) = 3.66, p = .06, which were superseded by an interaction of sample X singularity X identification, F(1,363) = 3.56, *p* = .06. Post hoc tests indicated WTC to identified single victim (M = 3.10) was higher than WTC to non-identified single victim (M = 2.32), for only American participants, *t*(78) = 3.01, *p* < .01. However, the single identified effect did not affect the WTC of Chinese sample, *t*(101) = 0.85, *p* = .40. Comparison of WTC within Chinese sample revealed greater mean scores for group condition (identified: M = 3.75, unidentified: M = 3.61) compared to single condition (identified: M = 2.92, unidentified: M = 3.12), *t*(103) = 3.67, *p* < .001, *t*(101) = 2.18, *p* = .03, for identified and unidentified types respectively, indicating that a group of victims received greater helping than a single victim for the Chinese participants, whether identified or not. In addition, the American sample also showed a similar pattern of helping a group only for unidentified condition, higher contributions were elicited when they faced an unidentified group than an unidentified individual (M = 2.99 vs. M = 2.32, *t*(80) = 2.60, *p* = .01) (presented in Fig 1A).

### The percentage of contributors

The percentage of contributors (people who contribute some amount as opposed to nothing) showed a similar trend as the amounts of WTC (presented in Fig 1B). For Chinese sample, the percentage of contributors to group was higher than single victim (97.1% vs. 93.2%, NS), and so was the American sample (90.4% vs. 81.2%, NS). More importantly, in the single victim condition, the percentage of American contributors to identified victim (90.0%) was significantly higher than unidentified victim (72.5%), (χ^2^ = 4.021, *df* = 1, *p* < .05). However, this pattern was not replicated in the group condition (*p* = .48) or in the Chinese sample (*p* = .30).

### Ratings of distress

Turning next to the ratings of affective reactions, the same 2 (sample) X 2 (singularity) X 2 (identification) analysis of variance (ANOVA) was also conducted as WTC. Participants experienced more distress when they faced a group than an individual, *F*(1,363) = 17.73, *p* < .001, that was superseded by an interaction of sample X singularity, *F*(1,363) = 4.67, *p* = .03, with post hoc tests indicating greater distress for a group (M = 4.60) compared to an individual (M = 3.35), for only Chinese participants, *t*(206) = 4.80, *p* < .001. However, the effect did not extended to the American sample, *t*(161) = 1.37, *p* = .17. In addition, there was no significant triple interaction between sample, identification and singularity in distress (*p* = .60) (Fig 1C).

### Ratings of sympathy

Similar as ratings of distress, participants felt greater sympathy when they faced a group than an individual, *F*(1,363) = 23.18, *p* < .001, that was superseded by an interaction of sample X singularity X identification, *F*(1,363) = 4.23, *p* = .04. Post hoc tests indicated that the identified single victim (*M* = 6.05) elicited considerably more sympathy than the non-identified single victim (*M* = 4.90), for only American participants, *t*(78) = 2.90, *p* < .01. However, the single identified effect did not affect sympathy of Chinese sample, *t*(101) = 0.25, *p* = .80. Comparison of sympathy within Chinese sample revealed greater scores for group condition (identified: M = 5.86, unidentified: M = 5.56) compared to single condition (identified: M = 4.07, unidentified: M = 4.16), *t*(103) = 5.16, *p* < .001, *t*(101) = 3.98, *p* < .001, for identified and unidentified types respectively, indicating that a group of victims received greater sympathy than a single victim for the Chinese participants, whether identified or not. In addition, in the unidentified condition, higher sympathy was evoked by American participants when they faced an unidentified group than an unidentified individual, which showed a clear tendency to significance (M = 5.67 vs. M = 4.90, *t*(80) = 1.96, *p* = .05) (Fig 1D).

### Emotions on WTC

Participants from both samples showed a significant correlation between ratings of distress and WTC, *r* = .46, *p* < .001 for the American participants and *r* = .33, *p* < .001 for the Chinese participants; as well as a significant correlation between ratings of sympathy and WTC, *r* = .50, *p* < .001 and *r* = .36, *p* < .001 for the American sample and the Chinese sample, respectively. It seemed that the higher the ratings of emotion the greater the willingness to contribute. In addition, we also found that the American participants’ ratings of distress significantly correlated with ratings of sympathy, r = .60, p < .001, and so did the Chinese participants, r = .63, p < .001.

To better understand the effect of evoked emotions on WTC, we analyzed Chinese participants' WTC by the two independent factors (singularity and identification), with ratings of distress and sympathy as covariates. Results still showed a significant main effect of singularity *F*(1,202) = 8.26, *p* < .01, and also significant effects of evoked affections for distress (*F*(1,202) = 4.21, *p* = .04), and sympathy (*F*(1,202) = 4.38, *p* = .04). Similarly, the same covariance analysis was also conducted in the American sample. Results only yielded highly significant effects of evoked emotions, for distress (*F*(1,157) = 8.10, *p* < .01) and sympathy (*F*(1,157) = 15.36, *p* < .001). In addition, there was no other indication reaching statistical significance.

## Discussion

The current study, examining Chinese and American participants, demonstrated the cultural differences in donation decision-making. The Chinese results revealed greater emotional reactions and WTC to a group of victims than an individual. While the American results suggested that a single identified victim elicited considerably higher contributions and sympathy than a non-identified individual, which were in line with the proposal that the identified victim effect is more pertinent for a single victim than for a group of victims [4,5,7,8].

It should be noted that the identifiable victim who has some information is more vivid than a statistical victim, the more vivid it is, the more sympathy is likely to be evoked [24]. And the identified information in the group may be not as vivid as it is in the single condition, which might lead to the less prominent effect in the group condition. Since Chinese participants preferred to help a group, the identified information did not play a prominent role in Chinese participants’ decisions, and the Chinese results did not duplicate the western findings that a single identified victim receives greater helping than a group of victims, whether identified or not. These results might in part be explained by the cultural differences in cognitive processing styles, the Chinese have been reported to attend less to focal objects than do the Americans [23], which might make the identified information less prominent.

The psychological mechanisms underlying the decision to help are different in Chinese and American cultures. Although the moral codes of both cultures encompass personal rights, personal duties and social goals, there is a difference in the priority given to the three concepts [25]. In contrast to the rights-based ethics of Western individualism, the duty-based ethics of Confucian relationalism in China provide a completely different perspective from Americans [19]. Chinese are more likely to emphasize social situations, whereas Americans are more likely to bias toward personal dispositions [26,27]. Moreover, it makes sense that the vivid information could exert an influence on personal feelings [4], but it seems to have nothing to do with moral responsibility, which might be a possible interpretation of the results that the identified victim effect is culture bound. This is also in line with our American results, the American participants also show a trend of helping the group, when there is no identifying information. In addition, the Chinese participants’ decisions demonstrated that a group of victims elicited greater emotional reactions and WTC than an individual. Since Confucian ethics in China emphasis on personal duties and social goals, helping more needy persons seems to be better related to the achievement of social goals. This is consistent with the rule of thumb in helping behaviors proposed by Mencius, take care of your aged parents first, and then extend your care to aged people in general; look after your own children first, and then extend to other children [19].

Previous research has highlighted the role of emotions in helping behaviors, and the emotional reactions guide the decision to help or not to help [9]. Indeed, our results indicated that the evoked emotions played an important role in both Chinese and American WTC. However, unlike the pattern found in American WTC, in which emotional reactions appeared to be the major source, Chinese preference for the group seemed to be somewhat independent of the emotional arousal toward the victims, at least, it did not totally depend on emotional factors. Previous studies showed that people's decision-making might accord to different social rules or norms. For example, Chinese might follow the reasonableness norm and integrate the considerations of both affective and rational factors when making a decision [28], and they are more inclined to prefer compromise and holistic resolutions [29,30]. In a recent study of moral decision-making task, Wang et al. [31] have also demonstrated that relative to Westerners, brain areas associated with attention, memory retrieval, and emotional processing were involved in Chinese, suggesting that Chinese tended to adopt a relatively integrated way of information processing.

In summary, our study indicates that it is important to consider cultural differences when trying to understand people's prosocial behavior. These different helping patterns between Chinese and American suggest that in order to optimally motivate people in situations where help is needed, culture must be taken into account. Future studies will be required to investigate the real monetary contributions and the underlying neural mechanism of cultural differences in helping decisions.

## Supporting Information

S1 Data Data used in the analysis. (XLSX)

## References

[1] Jenni K, Loewenstein G (1997) Explaining the identifiable victim effect. Journal of Risk and Uncertainty 14: 235–257.

[2] Schelling TC (1968) The life you save may be your own In Chase S. B. (Ed.), Problems in public expenditure analysis (pp. 127–162). Washington,DC: The Brookings Institution.

[3] Small DA, Loewenstein G (2003) Helping a victim or helping the victim: Altruism and identifiability. Journal of Risk and Uncertainty 26:5–16.

[4] Kogut T, Ritov I (2005) The "identified victim" effect: an identified group, or just a single individual? Journal of Behavioral Decision Making 18:157–167.

[5] Kogut T, Ritov I (2005) The singularity effect of identified victims in separate and joint evaluations. Organizational behavior and human decision processes 97:106–116.

[6] Ritov I, Kogut T (2011) Ally or adversary: The effect of identifiability in inter-group conflict situations. Organizational Behavior and Human Decision Processes 116(1):96–103.

[7] Kogut T, Ritov I (2007) "One of us": Outstanding willingness to help save a single identified compatriot. Organizational Behavior and Human Decision Processes 104(2):150–157.

[8] Small DA, Loewenstein G, Slovic P (2007) Sympathy and callousness: The impact of deliberative thought on donations to identifiable and statistical victims. Organizational Behavior and Human Decision Processes 102(2):143–153.

[9] Kogut T (2011) Someone to blame: When identifying a victim decreases helping. Journal of Experimental Social Psychology 47(4):748–755.

[10] Hernandez M, Iyengar SS (2001) What drives whom? A cultural perspective on human agency. Social Cognition 19:269–294.

[11] Markus HR, Kitayama S (1991) Culture and the self: Implications for cognition, emotion, and motivation. Psychological review 98(2):224–253.

[12] Kim Y, Sohn D, Choi SM (2011) Cultural difference in motivations for using social network sites: A comparative study of American and Korean college students. Computers in Human Behavior 27(1):365–372.

[13] Freeman JB, Ma Y, Han S, Ambady N (2013) Influences of culture and visual context on real-time social categorization. Journal of experimental social psychology 49(2):206–210. 23355750 10.1016/j.jesp.2012.10.015 PMC3551594

[14] Nisbett R E, Peng K, Choi I, Norenzayan A (2001) Culture and systems of thought: holistic versus analytic cognition. Psychological Review 108(2):291–310. 11381831 10.1037/0033-295x.108.2.291

[15] Nisbett RE, Masuda T (2003) Culture and point of view. Proceedings of the National Academy of Sciences of the United States of America 100(19):11163–11170. 12960375 10.1073/pnas.1934527100 PMC196945

[16] Barrett DW, Wosinska W, Butner J, Petrova P, Gornik-Durose M, Cialdini RB (2004) Individual differences in the motivation to comply across cultures: the impact of social obligation. Personality and individual differences 37(1):19–31.

[17] Levine RV, Norenzayan A, Philbrick K (2001) Cross-cultural differences in helping strangers. Journal of Cross-Cultural Psychology 32(5):543–560.

[18] Miller JG, Bersoff DM (1998) The role of liking in perceptions of the moral responsibility to help: A cultural perspective. Journal of Experimental Social Psychology 34(5):443–469.

[19] Bedford O, Hwang KK (2003) Guilt and Shame in Chinese Culture: A Cross-cultural Framework from the Perspective of Morality and Identity. Journal for the Theory of Social Behaviour 33(2):127–144.

[20] Federici S, Stella A, Dennis JL, Hünefeldt T (2011) West vs. West like East vs. West? A comparison between Italian and US American context sensitivity and Fear of Isolation. Cognitive processing 12(2):203–208. doi: 10.1007/s10339-010-0374-8 21063747 10.1007/s10339-010-0374-8

[21] Peng K, Nisbett RE (1999) Culture, dialectics, and reasoning about contradiction. American Psychologist 54(9):741–754.

[22] Kitayama S, Duffy S, Kawamura T, Larsen JT (2003) Perceiving an object and its context in different cultures. Psychological Science 14(3):201–206. 12741741 10.1111/1467-9280.02432

[23] Chua HF, Boland JE, Nisbett RE (2005) Cultural variation in eye movements during scene perception. Proceedings of the National Academy of Sciences of the United States of America 102(35):12629–12633. 16116075 10.1073/pnas.0506162102 PMC1194960

[24] Loewenstein G, Small DA (2007) The Scarecrow and the Tin Man: The vicissitudes of human sympathy and caring. Review of General Psychology 11(2):112–126.

[25] Dworkin R (1977) Taking rights seriously. Cambridge, MA: Harvard University Press.

[26] Menon T, Morris MW, Chiu C, Hong Y (1999) Culture and the construal of agency: Attribution to individual versus group dispositions. Journal of personality and social psychology 76(5):701–717.

[27] Morris MW, Peng K (1994) Culture and cause: American and Chinese attributions for social and physical events. Journal of Personality and Social psychology 67(6):949–971.

[28] Zhang Z, Yang C (1998) Beyond distributive justice: The reasonableness norm in Chinese reward allocation. Asian Journal of Social Psychology 1(3):253–269.

[29] Nisbett RE, Miyamoto Y (2005) The influence of culture: holistic versus analytic perception. Trends in Cognitive Sciences 9(10):467–473. 16129648 10.1016/j.tics.2005.08.004

[30] Miyamoto Y, Nisbett RE, Masuda T (2006) Culture and the physical environment. Psychological Science 17(2):113–119. 16466418 10.1111/j.1467-9280.2006.01673.x

[31] Wang Y, Deng YQ, Sui DN, Tang YY (2014) Neural correlates of cultural differences in moral decision-making: A combined ERP and sLORETA study. Neuroreport 25(2):110–116. doi: 10.1097/WNR.0000000000000077 24366325 10.1097/WNR.0000000000000077

